# An Original HPLC Method with Coulometric Detection to Monitor Hydroxyl Radical Generation via Fenton Chemistry

**DOI:** 10.3390/molecules24173066

**Published:** 2019-08-23

**Authors:** Maria Carmen Catapano, Michele Protti, Tommaso Fontana, Roberto Mandrioli, Přemysl Mladěnka, Laura Mercolini

**Affiliations:** 1 Department of Pharmacy and Biotechnology (FaBiT), Alma Mater Studiorum-University of Bologna, Via Belmeloro 6, 40126 Bologna, Italy; 2 Department of Pharmacology and Toxicology, Faculty of Pharmacy in Hradec Králové, Charles University, Heyrovského 1203, 50005 Hradec Králové, Czech Republic; 3 Department for Life Quality Studies (QuVi), Alma Mater Studiorum-University of Bologna, Corso d′Augusto 237, 47921 Rimini, Italy

**Keywords:** antioxidant, Fenton’s reaction, hydroxyl radical, HPLC-ED, coulometric detector, method validation

## Abstract

Hydroxyl radicals (•OH) can be generated via Fenton chemistry catalyzed by transition metals. An in vitro Fenton system was developed to test both the inhibition and stimulation of •OH formation, by monitoring salicylate aromatic hydroxylation derivatives as markers of •OH production. The reaction was optimized with either iron or copper, and target analytes were determined by means of an original HPLC method coupled to coulometric detection. The method granted good sensitivity and precision, while method applicability was tested on antioxidant compounds with and without chelating properties in different substance to metal ratios. This analytical approach shows how Fenton’s reaction can be monitored by HPLC coupled to coulometric detection, as a powerful tool for studying molecules′ redox behavior.

## 1. Introduction

Oxidative stress is defined as the lack of balance between the presence of chemically reactive oxidative species, particularly reactive oxygen species (ROS) and reactive nitrogen species (RNS), and the ability of the organism to counteract their action through its antioxidant protective systems, including both antioxidant enzymes and endogenous antioxidants. This redox balance can be broken by the presence of prooxidant factors (drugs and other xenobiotics, radiation, inflammation) [1] and/or through a decline in systems of protection from oxidation, leading to a significant decrease in the body capacity to contrast oxidative attacks directed toward biomolecular targets [2]. The above-mentioned processes are associated with aging and with some diseases, including carcinogenesis, hemochromatosis, Parkinson’s disease, Alzheimer’s disease, and Friedreich’s ataxia [3,4,5,6,7,8].

Antioxidants, in general, are present at low concentrations and react with ROS/RNS in order to neutralize their oxidative capacity. Antioxidants actually perform their activity through the release of electrons toward free radicals in order to increase the stability of the molecules themselves. However, it is well known that the body oxidative balance is also strongly connected to the presence and concentration of free transition metal ions (iron and copper), which can help generate ROS by reacting with hydrogen peroxide [9,10]. One of the main biological iron features is its ability to donate or receive an electron, i.e., to convert between its ferrous (Fe^2+^, Fe(II)) and ferric (Fe^3+^, Fe(III)) forms. However, this useful property for enzymatic redox reactions can be on the other hand harmful under some conditions, because iron is also known to generate ROS with relative ease [11,12,13,14]. Although less exploited, similar behavior is possible also for copper. Copper plays an important role in animal metabolism, in particular, because it allows many critical enzymes to function properly [15]. Copper antioxidant effects are linked principally to its presence in antioxidant enzymes (e.g., superoxide dismutase 1) [16]. On the other hand, it was suggested that its prooxidant activity via promotion of ROS production can contribute to the development of Alzheimer’s disease [17,18].

The most powerful ROS/RNS in biological systems is considered to be the hydroxyl radical (•OH). This radical reacts with most biological molecules, leading to the degradation of the molecules themselves with a series of chain reaction mechanisms [19,20,21,22]. It is produced mainly by the interaction of hydrogen peroxide (H_2_O_2_) with transition metals by the so-called Fenton’s reaction (Equation (1)):

M^n+^ + H_2_O_2_ → M^(n+1)+^ + OH^−^ + •OH (1)

The propagation of the reaction requires the recovery of the catalyst M^n+^, which can be pathophysiologically achieved by superoxide (Equation (2)):

M^(n+1)+^ + •O_2^−^_ → M^n+^ + O_2_ (2)

Hydroxyl radicals were first discovered in 1934 by Haber and Weiss [23,24] and the eponymous Haber–Weiss reaction (Equation (3)) summarizes the process (Equation (1) + Equation (2)):

(3) $$\begin{array}{c} •O_{2^{-}}\;+\;H_{2}O_{2}\;\overset{M^{n+}}{\to }\;•OH\;+\;OH^{-}\;+\;O_{2} \end{array}$$

The reaction is dependent on many factors, including availability of the catalyst (free iron or copper ions), pH value, presence of antioxidants, reductants, chelators, etc.

Bioactive molecules added to the reaction mixture may result in changes in the metal amount available to react with H_2_O_2_ and consequently in the production of •OH. The present study is focused on studying the effect of different known or possible antioxidants/chelators on the interaction between iron and copper ions and hydrogen peroxide to produce •OH. An antioxidant can either reduce the catalyst (e.g., behave as a superoxide in equation No. 2) or react directly with the formed hydroxyl radical or both. Most strong chelators can firmly bind iron and/or copper and hinder their catalytic activity, but there are even strong chelators like EDTA [25] which are not able to block the access of hydrogen peroxide to iron and which at the same time promote the Fenton chemistry by increasing the solubility of iron ions in the environment [26]. Hence the resulting effect (antioxidant, prooxidant or neutral) is theoretically hardly estimable and must be detected experimentally.

**Figure 1:**
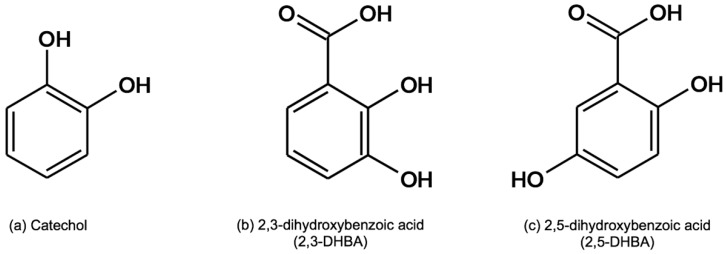
Salicylate hydroxylation products: catechol (**A**), 2,3-DHBA (**B**), and 2,5-DHBA (**C**).

At first, the Fenton’s reaction was triggered in order to obtain •OH generation. A sensitive and specific salicylate (2-hydroxybenzoic acid) hydroxylation assay was used [27] to monitor •OH production in conjunction with high-performance liquid chromatography with electrochemical detection (HPLC-ED), in particular by means of a coulometric detector. The products of •OH-mediated salicylate hydroxylation, which include 2,3- and 2,5-dihydroxybenzoic acid (2,3-DHBA and 2,5-DHBA) and catechol (Figure 1) [28], were detected and quantitated. The developed method was then used to evaluate the antioxidant and/or prooxidant activity of various bioactive molecules by comparing hydroxylation products (and thus •OH) formation rates in Fenton’s reaction mixtures with and without bioactive molecules.

Some published papers describe assays for the detection of •OH formation via Fenton’s reaction [20,27] and •OH production evaluation through HPLC-ED determination of hydroxylated salicylic acid [27,29,30,31,32] or tyrosine [33]. The method that uses tyrosine derivatives was, however, not characterized in detail (method validation results are mostly missing; method details are insufficient to allow replication) [33]. Among the papers that determine hydroxylated salicylic acid derivatives, some of them only include 2,3-DHBA and 2,5-DHBA [30,31,32]. Among those that include catechol as well [27,29,33], just A. Puppo et al. tested the •OH-scavenging effect of a narrow selection of compounds belonging to the class of flavonoids [33]; importantly, none of these papers included the study of Cu(I)-mediated Fenton’s reaction. Regarding the analytical technique, most published methods use amperometric detection, which is usually less selective and sensitive than coulometric detection [27,29,31,32,33]. Thomas et al. reported the use of a coulometric array detector, which can simultaneously monitor different detection potentials; however, no detail was included about the experimental conditions used for this application and no chromatograms were shown [30].

To the best of our knowledge, this is the first paper aiming at setting up a new, fully validated HPLC-ED method (with coulometric detector) to optimize Fenton’s reaction on both iron and copper salts, for studying both the antioxidant and prooxidant capacity of significant bioactive compounds.

## 2. Materials and Methods

### 2.1. Chemicals and Solutions

Catechol, 2,3-DHBA, 2,5-DHBA, salicylic acid, EDTA sodium salt (Na_2_EDTA), 2-hydroxybenzoic acid, 2,6-DHBA, 2,4-DHBA, sodium chloride (NaCl), copper chloride (CuCl), ferrous chloride (FeCl_2_), ferric chloride (FeCl_3_), and sodium hydrogen carbonate (NaHCO_3_), all in the form of pure powders (≥99%); quercetin (98% purity), catechin hydrate (≥98%), trientine (≥97%), EDTA (≥99%), phloroglucinol (≥99%), DOPAC (98%), 3-hydroxyphenylacetic acid (≥99%), homovanillic acid (HVA) (≥97%), and 5-chloro-7-iodo-8-hydroxyquinoline (≥95%); H_2_O_2_ (30%), orthophosphoric acid (H_3_PO_4_, 85%), hydrochloric acid (HCl, 37%), triethylamine (≥99.5%), methanol, and acetonitrile (HPLC grade, >99.9%) were purchased from Sigma-Aldrich (St. Louis, MO, USA). Ultrapure water, obtained from a Millipore Milli-Q system (Burlington, MA, USA), was used throughout the experiments.

Stock solutions (10 mM) of catechol, 2,3-DHBA, and 2,5-DHBA were prepared in ultrapure water. The corresponding working solutions were obtained by diluting stock solutions daily in ultrapure water. Stock solutions (10 mM) of the tested compounds were prepared in ultrapure water, except for quercetin and catechin stock solutions that were prepared in an ultrapure water/methanol (50/50, *V*/*V*) mixture. All stock solutions were stable for one week when stored at −20 °C; standard solutions were prepared fresh every day. All solutions were protected from light in amber glass vials from Waters (Milford, MA, USA).

### 2.2. Fenton’s Reaction Optimization

The formation of •OH via Fenton chemistry was monitored by the quantitation of three products: catechol, 2,3-DHBA, and 2,5-DHBA.

Fenton’s reaction in the presence of iron was triggered by adding 7.5 µL of 10 mM aqueous FeCl_3_ solution (or FeCl_2_ solution), 7.5 µL of 10 mM aqueous Na_2_EDTA, and 4 µL of 30% H_2_O_2_ to 940 µL of pH 7.40 bicarbonate solution (25 mM NaCl, 6.25 mM NaHCO_3_). This mixture was preincubated for 10 min at room temperature; then, 41 µL of 3 mM salicylic acid aqueous solution was added and the reaction was carried out for 2 min at room temperature. A volume of 200 µL of 4% phosphoric acid was added to stop the reaction. Immediately afterward, an aliquot of 100 μL of the solution was removed and transferred into a vial for HPLC-ED analysis.

The same procedures were implemented, both for FeCl_3_ and FeCl_2_ solutions, also without using Na_2_EDTA solution that was replaced with an equal volume of pH 7.40 bicarbonate solution.

For copper (10 mM aqueous CuCl solution), the reaction conditions and the procedure were identical, except for the Na_2_EDTA solution that was not used and was instead replaced by an equal volume of pH 7.40 bicarbonate solution.

Reaction optimization experiments were carried out using a mixture composed of 25 mM NaCl and 6.25 mM NaHCO_3_, where pH values ranging from 4.50 to 7.50 were studied to assess whether they could lead to variations in the •OH production. The assay of Fenton’s reaction was performed in the presence of either iron or copper ions.

Several tests regarding H_2_O_2_, in terms of both concentration and volume, were performed in order to optimize the Fenton chemistry. Preliminary tests were carried out by adding to the reaction mixture 2–5 μL of 3–30% H_2_O_2_ solution in water and observing the formation of the •OH-mediated salicylate hydroxylation products.

In order to monitor •OH generation, reaction time assays were performed. The amounts of catechol, 2,3-DHBA, and 2,5-DHBA produced at different reaction times (2, 5, 7, 10, 12, 15, 20, 25, and 30 min) were monitored and compared.

### 2.3. HPLC-ED Conditions

The chromatographic system consisted of a model PU-1580 pump from Jasco (Tokyo, Japan) with an ESA (Chelmsford, Massachusetts, MA, USA) coulometric Coulochem III detector, equipped with a high-sensitivity analytical cell with two porous graphite working electrodes placed in series in the same cell. The reference electrodes were α-hydrogen/palladium and the support electrodes were made of 501 stainless steel. In the analytical cell, potential 1 was set to −200 mV and potential 2 to +450 mV, with a range of 200 nA and a +1.00 V output. The coulometric detector was also equipped with a conditioning cell set to a potential of +50 mV. The chromatograms were acquired in oxidation mode. Electrodes were cleaned at the end of each working week in order to prolong their use time and to obtain reproducible results. All the electrodes were kept at +900 mV for 2 min to restore them, thoroughly oxidizing the components potentially fouling the electrodes.

A Waters Sunfire RP C18 column (100 × 4.6 mm, 3.5 μm), equipped with a Waters Sunfire guard column (4.6 mm, 3.5 μm), was used as the stationary phase. The mobile phase was a mixture of triethylammonium phosphate buffer (50 mM; pH 2.00) and acetonitrile (88/12, *V*/*V*). HPLC analysis was carried out in isocratic mode at a flow rate of 1.0 mL/min and using an injection volume of 50 μL. The data integration system was a DataApex (Petrzilkova, Prague, Czech Republic) Chromatography Station software (version CSW32 1.4).

### 2.4. System Suitability and Robustness

System suitability tests the overall performance of the analytical system considered as a whole and is an integral part of method development. Mean and percentage relative standard deviation (RSD%) values of retention time (RT), capacity factor (k), selectivity (α), resolution (R), total (N) and relative (N/m) efficiency and tailing factor (T_f_) were evaluated on six replicate injections of the analytes at a concentration of 100 nM each.

Robustness was assessed by evaluating the effect of small, deliberate changes to some key method parameters, namely: mobile phase buffer pH (1.8–2.2); mobile phase component ratio (85–90% buffer); flow rate (0.9–1.1 mL/min); injection volume (45–55 µL); temperature (18–28 °C); column lot. Fenton’s reaction and electrochemical detection conditions were not subjected to robustness tests since they had been already extensively tested during method development.

### 2.5. Method Validation

According to official guidelines [34,35], the following validation parameters were tested: linearity range, including limit of quantification (LOQ), limit of detection (LOD), precision, ruggedness, stability, and accuracy.

#### 2.5.1. Linearity

Calibration samples were prepared by adding known amounts of each analyte (catechol, 2,3-DHBA, and 2,5-DHBA) at six different concentrations to the blank matrix. The blank matrix was the non-triggered Fenton’s reaction medium, namely a mixture of pH 7.40 bicarbonate solution, H_2_O_2_, a transition metal (copper or iron), and EDTA. The resulting samples were injected into the HPLC-ED system. The procedure was carried out six times for each concentration. The obtained analyte peak areas were plotted against the corresponding nominal concentrations and the calibration curves set up by using the least-squares method. LOD and LOQ were computed on five calibration curves, using the following equations: 3.3 σ/S and 10 σ/S, respectively, where σ is the standard deviation of the intercept and S the slope of the calibration plot.

#### 2.5.2. Precision

Precision was evaluated by adding known amounts of the analytes (at six different concentrations, corresponding to lower limits, middle points, and a high value of each calibration curve) to the different types of blank matrices (with iron or copper) as defined above. The spiked samples were then subjected to HPLC-ED analysis. These assays were repeated six times within the same day to obtain intraday precision and six times over six different days to evaluate interday precision, in both cases expressed as percentage relative standard deviation (RSD%).

#### 2.5.3. Ruggedness and Stability

Ruggedness was evaluated by comparing the results of the analysis of the same quality control samples at three different concentrations (corresponding to lower limits, middle points, and high values of each calibration curve) obtained in two different laboratories (Bologna and Rimini), by four different analysts (two at each location), using two different instrumentations (HPLC instrumentations from different manufacturers, same detector model).

Analyte stability was assessed by analyzing the same samples (after Fenton’s reaction completion) in the following conditions: benchtop (24 h at room temperature); short-term refrigeration (1 week at 4 °C); long-term freezing (1 month at −20 °C) and freeze/thaw (3 freeze/thaw cycles over 3 weeks: one cycle consisted of freezing at −20 °C, then thawing at RT for 3 h, then freezing again, and was carried out at the end of each week).

#### 2.5.4. Accuracy

In order to evaluate method accuracy, after triggering, Fenton’s reaction was stopped at 2 min with phosphoric acid and an aliquot of the mixture was analyzed by HPLC-ED. Then, known amounts of the analytes at six concentrations of the calibration curve were added to other aliquots of the already analyzed samples. The assays were repeated six times during the same day to calculate the mean percentage recovery (%) of the added analytes and the corresponding RSD%.

### 2.6. Evaluation of the Anti- or Prooxidant Effect

Each substance to be evaluated was introduced into the reaction mixture at different substance:metal molar ratios, ranging from 1:10 to 10:1, replacing an equal volume of bicarbonate solution.

After being subjected to Fenton’s reaction in the presence of salicylic acid, the mixture was injected into the HPLC-ED system. The concentrations of the three analytes (catechol, 2,5-DHBA, and 2,3-DHBA) were determined separately by interpolation on the respective calibration curves and then added together; the obtained sum was compared to the sum of the analyte concentrations obtained without any tested compound. The result was expressed as the percentage of inhibition/induction of •OH production in comparison to that of the control sample not containing the tested compound.

## 3. Results and Discussion

### 3.1. Fenton’s Reaction

Firstly, an effective protocol for triggering Fenton’s reaction in the presence of iron or copper was developed. In addition to the metal ions, Fenton’s reaction requires the presence of bicarbonate, salicylic acid, and H_2_O_2_; EDTA can be added as well when using iron ions in particular to improve ferric ion solubility.

Several parameters were optimized, such as substrate concentration, bicarbonate solution pH, H_2_O_2_ concentration, and reaction time. These preliminary tests were carried out at the Bologna site laboratory.

#### 3.1.1. Salicylic Acid and Metal Ions

Salicylic acid was chosen as substrate because it easily undergoes radical hydroxylation and the products are detected by ED with high sensitivity. The three main hydroxylation products of salicylic acid are catechol, 2,3-DHBA, and 2,5-DHBA. Catechol originates from the aromatic radical addition of a hydroxyl group in position 1, followed by decarboxylation, while the other two products are derived by the same addition in other activated positions (3 and 5) [36,37]. These molecules are electroactive due to the vicinal phenol groups (catechol) that can be easily oxidized or reduced [38]. Preliminary tests were carried out to investigate the possible presence of secondary catechol products, such as 2,4-DHBA and 2,6-DHBA, and no detectable amount of either compound was found. In fact, the formation of these products is considered difficult because positions 4 and 6 of the aromatic ring of salicylic acid are deactivated and unavailable for a radical aromatic addition.

The influence of metal ion concentration was briefly studied over the 1–50 mM (added concentrations) range; the effect of concentration in this range on OH radical formation was not very large; however, 10 mM provided the highest formation rate and was thus chosen for further assays.

#### 3.1.2. Bicarbonate Solution pH

Experiments were performed at five (patho-) physiologically relevant pH values of the bicarbonate solution: 4.50, 5.50, 6.80, 7.40, and 7.50. In the presence of both iron and copper, altering the pH value produces a change in •OH formation: •OH production decreases progressively going toward acidic pH values. The best signal to noise ratio was obtained working at pH 7.4, which is also the physiological pH of plasma, and for this reason, it was chosen to perform the following experiments.

#### 3.1.3. Hydrogen Peroxide Concentration and Volume

H_2_O_2_ was studied in terms of concentration (3–30%) and volume (2–5 μL) in order to optimize the Fenton chemistry. Preliminary tests were carried out adding 2 μL of 3% H_2_O_2_ to the reaction mixture, but analyte formation was detected only in negligible amounts. In order to increase the extent of their production, the amount of H_2_O_2_ and its concentration were gradually increased and a volume of 4 μL of 30% allowed to reach the optimal signal of the three hydroxylation products.

#### 3.1.4. Reaction Time

Incubation times before triggering Fenton’s reaction were evaluated in the 5–30 min range. A 10-min incubation time was chosen because longer times did not provide better sensitivity.

In order to monitor •OH generation, time-dependent kinetic assays were performed. The amount of analytes produced at different reaction times (from 2 to 30 min) was monitored and compared: after just 2 min, the production of the three phenolic compounds was already almost complete. This allows to obtain reliable results in a short time. So, it was decided to stop Fenton’s reaction by adding phosphoric acid after 2 min in all subsequent assays.

### 3.2. HPLC-ED Analysis

**Figure 2:**
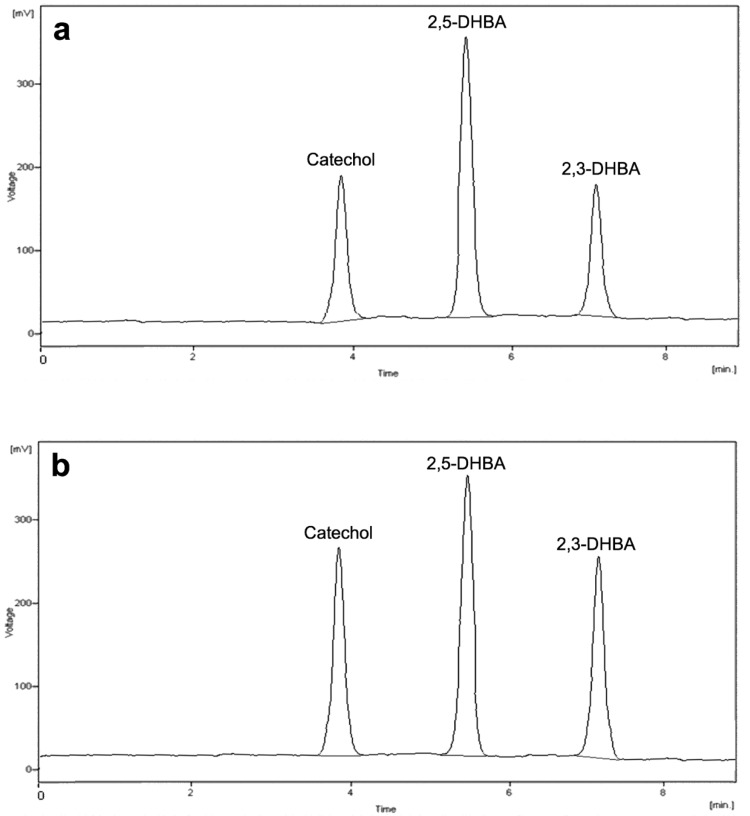
Chromatograms of salicylic acid hydroxylation products after carrying out a standardized Fenton’s reaction in the presence of iron, Fe(III) (**a**) and copper, Cu(I) (**b**).

The salicylate hydroxylation assay with production of catechol, 2,5-DHBA, and 2,3-DHBA was monitored by HPLC-ED (coulometric detector) to assess •OH generation. In Figure 2, chromatograms of salicylic acid hydroxylation products after the standardized Fenton’s reaction in the presence of Fe(III) (a) and Cu(I) (b) are shown.

The use of coulometric detection enhances the sensitivity and selectivity of the HPLC method [38,39], thanks to the specific cell geometry allowing consistent and extensive compound oxidation (or reduction). Most published papers on the HPLC-ED analysis of hydroxylated salicylic acid derivatives reported the use of amperometric detection instead, which is less selective and sensitive than coulometric detection [27,29,31,32,33]. Thomas et al. reported the use of a coulometric array detector, but the paper lacks any analytical conditions and performance details [30].

First of all, the potentials to be applied to the analytical cells were studied. Screening assays were carried out by varying E1 and E2 potential values to optimize the working conditions. For compounds that are reversibly oxidable, E1 is often set at a negative or slightly positive potential to keep the analytes reduced, while E2 is set at a positive potential strong enough to completely oxidize the analytes, thus obtain the highest sensitivity. E1 was varied between −500 and +200 mV, while E2 was varied between +100 and +600 mV. When E1 was lower than −400 mV, strong interference was detected (presumably due to oxygen oxidation) and when it was higher than +100 mV, 2,3-DHBA and 2,5-DHBA peaks were not detected (possibly due to their fast oxidation). E1 was thus set at −200 mV to grant good selectivity and sensitivity. Regarding E2, method sensitivity increased almost proportionally when increasing the potential; however, at high potential values (at +500 mV and above), baseline instability and interferences occurred. +450 mV was chosen as the best compromise for E2.

The conditioning cell was set to a weak oxidation potential of +50 mV, to irreversibly oxidize interferents with a low standard reduction potential and further increase the selectivity of the method.

**Figure 3:**
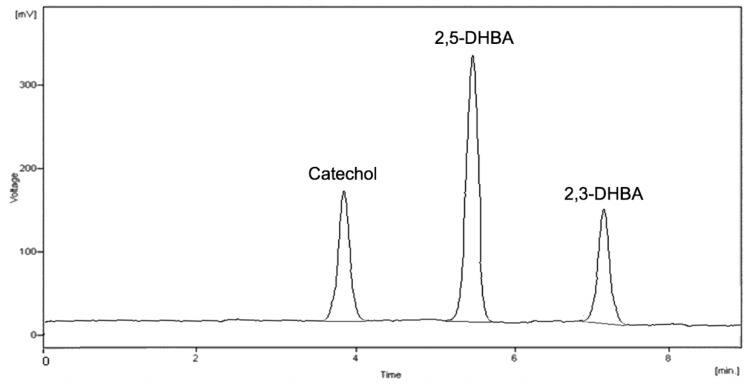
Chromatogram of salicylic acid hydroxylation products after carrying out a standardized Fenton’s reaction in the presence of iron, Fe(II).

The best reversed-phase chromatographic conditions were also studied. Bonded-silica C8 and C18 stationary phases were tested and the best results were obtained on a C18 column (100 × 4.6 mm, 3.5 μm) with high mass loading capability and excellent pH stability, granting good peak shape and efficiency. For mobile phase preparation, different buffers were tested (phosphate, acetate, formate) in the 2–7 pH range. It was ascertained that peak separation increased by decreasing the pH of the buffer, probably due to better retention of the undissociated acids. For this reason, phosphate buffer at pH 2.0 was deemed appropriate and the most effective in the working conditions. A quite low percentage (12%) of acetonitrile added for mobile phase preparation was sufficient for satisfactory analyte resolution within acceptable run times; a complete analytical run with baseline separation lasted 8 min, as can be seen from Figure 3 where a chromatogram of salicylic acid hydroxylation products after carrying out the standardized Fenton’s reaction in the presence of Fe(II) is reported.

The peak area ratios among the three analytes were not perfectly constant; as a result, proportionality between concentration and sum of analyte peak areas was much better than proportionality between concentration and any single analyte peak area. The former was thus chosen for quantitative purposes.

### 3.3. System Suitability and Robustness

After standardization of the analytical conditions, extensive system suitability assays were carried out. The complete list of results and their comparison with commonly accepted specifications are reported in Table S1. As can be seen, all parameters are well within specifications. Thus, method suitability is good.

The results of robustness assays are reported in Table S2. The effects of every change on system suitability parameters are well within reasonable limits, with good proportionality within the tested ranges. Thus, the effects are quite predictable, and these changes should not significantly impact on the reliability of analytical results.

### 3.4. Method Validation

In Table 1 it is possible to observe the data for the quantitative determination of the reaction products obtained by the standardized Fenton’s reaction in the presence of iron and copper.

The concentrations of the three analytes catechol, 2,3-DHBA, and 2,5-DHBA are expressed as mean values after six repetitions over six different days. From these results, it appears that Cu ions are more efficient catalysts of the Fenton Reaction than both oxidation states of Fe. More assays are in progress in order to test this hypothesis.

#### 3.4.1. Linearity and Precision

Linearity range was tested on six different concentration points. Satisfactory linearity (r^2^ > 0.9990) was obtained for all the three analytes within their concentration ranges (see Table 2). For each individual compound, LOD and LOQ values were also determined, demonstrating a very high sensitivity of the analytical method. The detailed results are reported in Table 2.

Precision was evaluated over six repetitions at three concentration levels. The resulting data provided good precision, with RSD values always lower than 4.5% (Table 3). For comparison, U.S. Food and Drug Administration’s (FDA’s) acceptance criterion for precision is RSD < 15% for all concentration levels, except RSD < 20% for the low concentration level [35].

#### 3.4.2. Ruggedness and Stability

Method ruggedness was evaluated as the resulting variability caused by different analysts, working on different equipment and in different laboratories. The complete assay results are shown in Table S3. As can be seen, RSD% was always lower than 10% and mean bias was always lower than 9%, both well within acceptable limits.

Analyte stability after Fenton’s reaction was assessed under different conditions, and the corresponding results are reported in Table S4. Stability was good, or at least acceptable, under all conditions tested; in fact, the maximum analyte loss of 13% was observed for benchtop stability samples after 4 h at RT (acceptance criterion: loss < 15% [35]). Since benchtop stability samples showed a trend toward progressive analyte degradation, it is suggested that all samples should be analyzed within 1 h from the preparation, or else be kept frozen, and thawed just a few minutes before analysis to ensure optimal analysis reliability.

#### 3.4.3. Accuracy

Good mean percentage recovery was obtained for accuracy (>92%). The corresponding RSD was calculated over six repetitions, proving a very good precision, always lower than 4.1%.

### 3.5. Application of Fenton’s Reaction to Bioactive Molecules

The introduction of different bioactive substances with antioxidant and/or chelating properties into the reaction mixture clearly influenced the amount of •OH production, which in turn resulted in changes in the three measured product concentrations. A decrease in the production of catechol, 2,3-DHBA, and 2,5-DHBA indicates a corresponding proportional decrease in •OH production and is consequently related to the antioxidant activity of the substance. Conversely, an increase in the production of the three analytes indicates an increase in the generation of •OH and therefore a prooxidant activity of the molecule under investigation. It is also possible that the presence of the substance does not change the analyte concentrations.

Analyses were carried out by adding each substance under examination to the optimized reaction mixture, testing molar substance:metal ratios between 1:10 and 10:1. The tested substances were: 3-hydroxyphenylacetic acid, 3,4-dihydroxyphenylacetic acid (DOPAC), 5-chloro-7-iodo-8-hydroxyquinoline, catechin, EDTA (only for copper), homovanillic acid (HVA), quercetin, phloroglucinol, and trientine (only for copper). The selection of the compounds was based on their known different properties: iron or copper chelators without iron/copper reducing activity (EDTA, trientine, 5-chloro-7-iodo-8-hydroxyquinoline), chelators with iron/copper reducing activity (quercetin, DOPAC and catechin), pure antioxidants without chelating properties (phloroglucinol, homovanilic acid and 3-hydroxyphenylacetic acid).

The obtained results are reported in Table 4. Both antioxidant and prooxidant properties were reported. These were largely concentration-dependent. In fact, the percentage of •OH formation was always the lowest at the highest substance concentrations (e.g., 0% •OH formation at 10:1 DOPAC:metal ratio with Fe(II)) and the highest at the lowest substance concentration (e.g., 280% •OH formation at 1:10 DOPAC:metal ratio with Cu(I)).

However, significant differences were noted between iron and copper reactions. All tested substances were quite effective in inhibiting •OH formation (0–4% •OH formation) in the iron systems at high substance:metal ratios, with the antioxidant effect decreasing moderately (up to 25% •OH formation) with the ratio.

HVA seemed to be the only compound to lose its antioxidant power altogether when diluted (51%, 100% •OH formation), despite being one of the most active compounds at high ratios (together with 3-hydroxyphenylacetic acid and DOPAC).

In the copper system, most compounds apart from DOPAC and phloroglucinol showed good antioxidant power (>75% inhibition) at high substance:metal ratios, but all lost their activity and even became prooxidant (•OH formation >100%) at lower ratios. Globally, in the copper system, DOPAC possessed the least antioxidant activity, while the most effective ones seemed to be HVA and trientine.

The tested compounds could act as antioxidants either by directly reacting with •OH (they would compete with salicylic acid) or by decreasing •OH formation; it is currently impossible to discriminate these two mechanisms with this analytical methodology. However, most compounds become prooxidant at low substance:Cu ratios, so the most probable mechanism is recovery of the catalyst by reduction of Cu^2+^ ions.

## 4. Conclusions

An optimized salicylate hydroxylation assay coupled to an original HPLC-ED (coulometric detector) method was used to monitor •OH production within the Fenton’s reaction. By using this type of instrumental method, it was possible to give high sensitivity and selectivity to the analysis, reliably determining low concentrations of the three reaction products represented by catechol, 2,3-DHBA, and 2,5-DHBA. The analytical method was first validated obtaining excellent results in terms of linearity, precision, and accuracy. After validation, the method was applied to monitoring the Fenton’s reaction for the study of known or prospective antioxidant substances of natural and synthetic origin and iron/copper chelators. This method confirms that the redox behavior of these bioactive molecules can be at least in part related to their capacity to block or increase •OH production via Fenton chemistry.

Body oxidative balance is strongly connected to both antioxidant compounds acting through the release of electrons able to stabilize toward free radicals, as well as to the presence and concentration of free transition metal ions (iron and copper), able to produce ROS by interacting with hydrogen peroxide via the Fenton’s reaction. More quantitative assays, particularly related to more detailed concentration–effect curves and wider substance:metal ratio ranges, are underway to confirm or refute the results obtained in this research work. More studies are also needed to verify whether part of the antioxidant effect is in any way related to the metal chelating properties of the compounds. In any case, the developed analytical method demonstrated an effective and promising tool for determining the antioxidant or prooxidant effect of numerous, chemically different compounds, also paving the way for more detailed studies on the antioxidant and prooxidant mechanism of action of in-vivo complex systems.

## Figures and Tables

**Table 1:** Concentrations of the Three Hydroxylation Products Obtained by Fenton’s Reaction in the Presence of Iron or Copper.

Fenton’s Reaction	Catechol Concentration (mean ± SD, nM) ^a^	2,3-DHBA Concentration (mean ± SD, nM) ^a^	2,5-DHBA Concentration (mean ± SD, nM) ^a^
**Iron, Fe(III)**	59.0 ± 2.1	139.3 ± 3.0	135.0 ± 2.8
**Iron, Fe(II)**	63.9 ± 1.7	133.4 ± 2.8	130.1 ± 3.3
**Copper, Cu(I)**	200.1 ± 5.1	385.5 ± 8.3	149.3 ± 3.9

^a^ Mean concentrations and SD values obtained from six repetitions over six different days.

**Table 2:** Method Linearity.

Analyte	Metal	Linearity Range (nM)	r^2^	LOD (nM)	LOQ (nM)
**Catechol**	Fe(III)	9.0–450	0.9994	3.0	9.0
	Fe(II)	9.0–450	0.9993	3.0	9.0
	Cu(I)	3.0–450	0.9992	1.0	3.0
**2,3-DHBA**	Fe(III)	6.5–325	0.9998	2.0	6.5
	Fe(II)	6.5–325	0.9991	2.0	6.5
	Cu(I)	6.5–325	0.9995	2.0	6.5
**2,5-DHBA**	Fe(III)	6.5–325	0.9996	2.0	6.5
	Fe(II)	6.5–325	0.9997	2.0	6.5
	Cu(I)	2.0–325	0.9994	0.7	2.0

**Table 3:** Method Precision.

Analyte	Metal	Concentration (nM)	Intraday Precision (RSD%) ^a^	Interday Precision (RSD%) ^a^
**Catechol**		9	3.8	4.1
	Fe(III)	200	2.7	3.1
		450	2.3	2.9
		9	3.6	3.5
	Fe(II)	200	2.8	3.0
		450	2.5	2.9
		3	1.4	1.7
	Cu(I)	200	0.9	1.3
		450	0.7	1.2
**2,3-DHBA**		7	2.6	3.9
	Fe(III)	150	1.6	2.2
		325	1.3	2.0
		7	1.2	3.1
	Fe(II)	150	0.5	2.1
		325	0.4	1.7
		7	1.4	3.0
	Cu(I)	150	0.5	1.4
		325	0.4	1.0
**2,5-DHBA**		7	4.2	4.3
	Fe(III)	150	3.0	3.3
		325	2.9	3.1
		7	2.7	4.4
	Fe(II)	150	2.2	3.1
		325	2.0	2.6
		2	1.4	1.6
	Cu(I)	150	1.0	1.4
		325	0.9	1.3

^a^ n = 6.

**Table 4:** Effect of the Tested Compounds on the Fenton Reaction.

Compound	Range of •OH Formation (%) ^a^		
	Iron (II)	Iron (III)	Copper (I)
3-Hydroxyphenylacetic acid	0–22	0–21	6–131
3,4-Dihydroxyphenylacetic acid (DOPAC)	0–13	0–11	64–280
5-Chloro-7-iodo-8-hydroxyquinoline	4–17	3–23	16–131
Catechin	2–12	2–11	23–127
EDTA ^b^	-	-	3–113
Homovanillic acid (HVA)	1–51	0–100	0–174
Quercetin	3–21	3–21	14–136
Phloroglucinol	3–20	2–25	44–149
Trientine ^b^	-	-	1–128

^a^ Calculated as: (amount of •OH formed with the compound/amount of •OH formed without the compound) × 100; *n* = 3. Higher values correspond to lower antioxidant powers; values higher than 100% correspond to prooxidant activities. ^b^ EDTA and trientine were not tested as antioxidants with iron.

## Supplementary Materials

The following are available online, Table S1. System suitability data and specification limits, Table S2. Results of robustness tests, Table S3. Ruggedness assay results, Table S4. Results of stability assays.
