# Supplementary material for: An Original HPLC Method with Coulometric Detection to Monitor Hydroxyl Radical Generation via Fenton Chemistry
Source: Molecules. 2019 Aug 23;24(17):3066. doi: 10.3390/molecules24173066 (PMC6749383; doi:10.3390/molecules24173066)
Supplement: Supplementary file 1 [file molecules-24-03066-s001.pdf]

Supplementary Materials

An original HPLC method with coulometric detection to monitor hydroxyl radical generation via Fenton chemistry

Maria Carmen Catapano[1,2], Michele Protti[1*], Tommaso Fontana[1], Roberto Mandrioli[3], Přemysl Mladěnka[2] and Laura Mercolini[1]

[1] Department of Pharmacy and Biotechnology (FaBiT), Alma Mater Studiorum - University of Bologna, Via Belmeloro 6, 40126 Bologna (Italy).

[2] Department of Pharmacology and Toxicology, Faculty of Pharmacy in Hradec Králové, Charles University, Heyrovského 1203, 500 05 Hradec Králové (Czech Republic).

[3] Department for Life Quality Studies (QuVi), Alma Mater Studiorum - University of Bologna, Corso d'Augusto 237, 47921 Rimini (Italy).

* Correspondence: michele.protti2@unibo.it; Tel.: +39-051-209-9740

Table S1. System suitability data and specification limits.

Parameter	Analyte	Mean[a,b]	RSD%[a]	Specification
Retention (t_R), min	Catechol	3.92	0.3	RSD% < 2
	2,5-DHBA	5.38	0.3	
	2,3-DHBA	7.12	0.4	
Capacity (k)	Catechol	2.02	0.3	1.5 < k < 5
	2,5-DHBA	3.29	0.3	
	2,3-DHBA	4.54	0.4	
Selectivity (α)	Catechol / 2,5-DHBA	1.63	0.4	α > 1.1
	2,5-DHBA / 2,3-DHBA	1.38	0.4	
Resolution (R)	Catechol / 2,5-DHBA	5.54	0.4	R > 2
	2,5-DHBA / 2,3-DHBA	4.61	0.4	
Efficiency (N)	Catechol	3400	0.7	N > 2500
	2,5-DHBA	4700	0.8	
	2,3-DHBA	5800	0.8	
HETP (H), µm	Catechol	30	0.7	H < 40
	2,5-DHBA	21	0.8	
	2,3-DHBA	17	0.8	
Peak asymmetry (A_s)	Catechol	1.08	1.0	0.8 < A_s < 1.25
	2,5-DHBA	1.02	0.9	
	2,3-DHBA	0.91	1.1	

[a] $n = 6$.

Table S2. Results of robustness tests.

Parameter	Range	Performance index[a]	Effect (variation range)[b]
Buffer pH	1.8 - 2-2	k	-9% to + 15%
		R	-8% to +12%
		N	-2% to +5%
		T_f	-0.1% to +0.2%
Mobile phase composition	85 - 90% buffer	k	-15% to + 20%
		R	-10% to -17%
		N	-5% to +1%
		T_f	+5% to +16%
Flow rate	0.9 - 1.1 mL/min	k	-10% to + 13%
		R	-0.5% to +1%
		N	-0.5% to +0.7%
		T_f	+5% to +9%
Injection volume	45 - 55 μL	k	-0.0% to + 0.1%
		R	-6% to +3%
		N	-0.1% to +0.1%
		T_f	-0.0% to +10%
Temperature	18 -28°C	k	-17% to + 13%
		R	-5% to +4%
		N	-12% to +7%
		T_f	-0.0% to +0.1%
Column production lot	3 different lots	k	-0.4% to + 0.5%
		R	-0.0% to +3%
		N	-3% to +5%
		T_f	-0.2% to +0.1%

[a] k = capacity factor; R = resolution; N = theoretical plate number; T_f = tailing factor.
[b] n = 6.

Table S3. Ruggedness assay results.

Variable			Concentration	Mean bias (%)[a]	RSD%[a]
**Analyst 1**	**Instrument 1**	Location 1	Low	-	-
			Middle	-	-
			High	-	-
	Instrument 2	Location 2	Low	-4.0	5.2
			Middle	-3.1	3.9
			High	+1.7	2.8
Analyst 2	Instrument 1	Location 1	Low	-5.7	8.4
			Middle	-2.2	6.4
			High	-1.6	4.0
Analyst 3	Instrument 1	Location 1	Low	+6.9	7.0
			Middle	+3.4	5.2
			High	+2.0	3.1
	Instrument 2	Location 2	Low	-5.6	6.2
			Middle	-4.1	5.6
			High	-2.9	4.2
Analyst 4	Instrument 2	Location 2	Low	-6.7	6.8
			Middle	+3.9	6.3
			High	+2.8	4.3

[a] The results are relative to the baseline of Analyst 1 working on Instrument 1 at Location 1.

Table S4. Results of stability assays.

Type	Temperature	Time	Concentration	% Recovery
Benchtop	RT	4 h	Low	96.5
			High	97.0
		12 h	Low	91.4
			High	93.7
		24 h	Low	86.8
			High	87.1
Refrigerated	4°C	1 d	Low	97.1
			High	97.8
		3 d	Low	94.0
			High	95.1
		7 d	Low	91.3
			High	91.7
Frozen	-20°C	7 d	Low	99.3
			High	99.5
		15 d	Low	98.4
			High	99.0
		30 d	Low	97.6
			High	98.2
Freeze-thaw	-20°C	3 cycles: 7, 14, 21 d	Low	95.3
			High	96.8
